# In Vivo Optical Coherence Tomography for the Detection, Subtyping, and Margin Assessment of Facial Basal Cell Carcinoma: A Comparative Study with Histopathology

**DOI:** 10.3390/jcm14030949

**Published:** 2025-02-01

**Authors:** Waseem Jerjes, Zaid Hamdoon, Dara Rashed, Colin Hopper

**Affiliations:** 1 Faculty of Medicine, Imperial College London, London SW7 5NH, UK; 2 Research and Development Unit, Hammersmith and Fulham Primary Care Network, London W6 7HY, UK; 3 College of Dental Medicine, University of Sharjah, Sharjah P.O. Box 27272, United Arab Emirates; zothman@sharjah.ac.ae; 4 Unit of OMFS, UCL Eastman Dental Institute, London WC1E 6DE, UK; d.rashed@alumni.ucl.ac.uk (D.R.); c.hopper@ucl.ac.uk (C.H.)

**Keywords:** basal cell carcinoma, skin cancer, OCT, BCC, facial lesions, optical coherence tomography

## Abstract

**Background:** Basal cell carcinoma (BCC) is the most common skin cancer, with several subtypes that vary from one another in biological behaviour and therapeutic consequences. The aim of the study was to evaluate in vivo OCT diagnostic accuracy to detect and subtype facial BCCs, using histopathology as the gold standard. **Patients and Methods:** This single-centre, prospective, diagnostic accuracy study was carried out on 136 patients who were presenting with a total of 220 clinically suspicious facial lesions for BCC. All lesions were imaged by OCT before their surgical excision. OCT findings were compared to the histopathological results in the respect of detection of BCC, subtype, tumour depth, and margin status. **Results:** OCT showed an excellent diagnostic performance for the detection of BCC in general: sensitivity, 96.8%; specificity, 98.2%; and accuracy, 97.5%. The AUC for the detection of BCC was 0.97. Subtype sensitivity for OCT was highest for superficial, 93.1%, and nodular BCC, 92.1%, and marginally lower for micronodular, 89.3%, and infiltrative subtypes, 90.0%. Depth measurements of OCT-derived tumour strongly correlated with those obtained by histopathology: mean depth, 2.3 ± 0.9 mm for OCT versus 2.2 ± 0.8 mm for histopathology; *p* = 0.08. **Conclusions:** The performed OCT showed very good agreement in the detection, subtyping, and preoperative assessment of BCC facial lesions. In addition to its non-invasive characteristics, its robustness regarding the evaluation of tumour depth and margins makes OCT very promising for improved surgical planning by reducing unnecessary excisions.

## 1. Introduction

Basal cell carcinoma (BCC) is the most common form of skin cancer worldwide, accounting for about 80% of all non-melanoma skin cancers. BCC arises mainly from basal keratinocytes within the epidermis due to chronic exposure to ultraviolet radiation, especially in fair-skinned individuals with high lifetime UV exposure [1]. The incidence of BCC is rising because of increased sun exposure, the aging of the population, and improved diagnostic techniques and, therefore, is considered to be an emerging public health concern [2]. Although metastasis rarely occurs in cases of BCC, significant clinical problems are caused by its local invasiveness and tissue destruction, especially when it appears in cosmetically sensitive facial areas [3].

Clinically, BCCs can present with diverse morphologies, and skin lesions usually present as pearly nodules, ulcerated plaques, or pigmented areas. Because of such variability, BCCs are histologically classified into superficial, nodular, micronodular, infiltrative, and mixed subtypes, each of which has different biological behaviours and treatment implications [4]. The nodular BCC is the most common variant, but infiltrative and micronodular subtypes are more aggressive and tend to recur. The mainstays in minimizing morbidity and achieving optimal outcomes in treatment involve early diagnosis and precise preoperative assessment [5].

Traditionally, histopathological examination after biopsy or surgical excision has been the gold standard for diagnosing and subtyping BCCs. However, histopathology is invasive, time-consuming, and lacks the ability to provide real-time diagnostic information. Recently, non-invasive imaging techniques have emerged as useful tools for preoperative BCC assessment. Among these, Optical Coherence Tomography (OCT) has gained prominence due to its capability to produce high-resolution, cross-sectional imaging of skin layers in real time [6]. OCT uses near-infrared light to generate images of the epidermis, dermoepidermal junction, and superficial dermis, allowing clinicians to assess lesion depth, margins, and morphology without requiring tissue excision [7].

OCT has shown superior diagnostic capability compared to standard dermoscopy in identifying and subtyping basal cell carcinoma (BCC). While dermoscopy is limited to surface visualization, OCT provides cross-sectional imaging that enables real-time assessment of tumour depth, margin involvement, and subtypes. This makes OCT a valuable tool for improving preoperative planning and minimizing unnecessary biopsies [8,9].

Several studies have explored one or another diagnostic capability of OCT methodology in the detection of BCC, and reported sensitivities and specificities were more than 90 percent for the identification of BCC lesions compared with histopathology examination/investigation [8]. Indeed, good diagnosis with OCT for separation among BCC subvarieties is achieved on conventional imaging features of varieties of BCC, which include, among others, hyporeflective nests, elongated cable-like strands, and dermis junction disruption. Superficial BCCs are characterized by hyporeflective nests confined to the upper dermis, while nodular BCCs appear as well-circumscribed, round nests with palisading edges extending into the mid-dermis [9]. The more aggressive variants, like infiltrative BCCs, present as thin, irregular hyporeflective strands that infiltrate deeper into the dermis; thus, their detection becomes highly crucial for treatment planning [10].

Nevertheless, the clinical utility of OCT, in regard to exact demarcation of tumour margins and depth estimation with high accuracy, continues to remain under evaluation because surgical decisions highly depend on it. Establishment of proper margins will also prevent recurrence with a minimum sacrifice of normal tissues surrounding the tumour. Reliable measurement of tumour depth and margins can be performed by OCT for pre-surgical planning that enables reduction of re-excisions, which can ultimately bring about improved outcomes in the patient. Moreover, the inter-observer reproducibility of OCT results is an integral part of this technique entering regular clinical practice.

This study has been designed to assess the diagnostic accuracy of in vivo OCT for the detection and subtyping of facial BCCs against histopathology as a gold standard. Furthermore, we aimed at evaluating the capability of OCT in the preoperative demarcation of tumour margins and measuring the depth of lesions. Therefore, comparison of OCT findings to histopathology across a broad patient cohort will be enlightening in terms of its clinical utility as a non-invasive diagnostic tool for BCC management.

## 2. Patients and Methods

### 2.1. Study Design and Participants

This is a prospective, single-centre observational diagnostic accuracy study conducted in a tertiary care centre (UCLH Head and Neck Centre) that specializes in surgical oncology. The primary aim of the study was to assess the diagnostic performance of in vivo Optical Coherence Tomography (OCT) in detecting and subtyping facial basal cell carcinomas (BCCs), using histopathology as the gold standard. A secondary objective was to evaluate the accuracy of OCT in delineating tumour margins prior to surgery. The study was conducted according to the guidelines of the Declaration of Helsinki, and approved by the Moorfields and Whittington Local Research Ethics Committee of the National Health Service England (REC reference number 07/Q0504/4, protocol code 1 and date of approval 21 February 2017), and all participants provided written informed consent before being included in the study. This study employed a non-probabilistic convenience sampling method to recruit participants.

Eligibility criteria required adult patients, 18 years or older, presenting with one or more untreated lesions localized to the face. Lesions were assessed as clinically suggestive of BCC based on macroscopic features, including pearly borders, telangiectasia, pigmentation, ulceration, or surface scaling. Patients with recurrent or previously treated BCCs, non-facial lesions, or lesions considered too superficial—defined as lesions with minimal thickness and confined to the superficial epidermis, where imaging penetration and excision would be insufficient—were excluded.

At the first visit itself, all lesion characteristics were meticulously noted and recorded. Calibrated digital callipers measured the lesion size by precise measurement of length, width, and height. The lesions were grouped by their anatomical distribution over the face to investigate possible clinical presentations. Each lesion was characterized as symptomatic, including pain, bleeding, and itching, or asymptomatic.

Where the suspicion of BCC was not as clear, alternative diagnoses were considered for lesions. The clinical differentials included actinic keratosis, seborrheic keratosis, squamous cell carcinoma, and benign lesions including intradermal nevi.

### 2.2. Imaging Protocol

All the lesions were subjected to in vivo OCT imaging before surgical excision. Scans were performed with the VivoSight^®^ OCT system (Version 1) from Michelson Diagnostics, Maidstone (UK), a high-resolution dermatological OCT device that uses a lateral resolution of 7.5 μm and an axial resolution of 5 μm. Near-infrared light at a wavelength of 1305 nm was used to obtain detailed en face and cross-sectional images of the epidermis, DEJ, and upper dermis.

Each lesion was scanned in a systematic fashion in the *X*-*Y*-*Z* axes to capture comprehensive three-dimensional data of tumour depth, width, and margins. Scanning extended 5 mm beyond the clinically visible borders of lesions to assess surrounding tissue and facilitate preoperative margin evaluation. Imaging sessions were completed in approximately 5 min per lesion. OCT data were anonymized and reviewed offline for quality assurance. Operators were blinded to histopathological findings to minimize bias.

### 2.3. Surgical Excision and Histopathology

Subsequently, after imaging, lesions were surgically excised under local anaesthesia using standardized elliptical margins of 5 mm. The excised specimens were oriented carefully, labelled, and inked to facilitate an accurate histopathological assessment. Tissue specimens were fixed in 10% buffered formalin, embedded in paraffin, sectioned at 4 μm thickness, and stained with haematoxylin and eosin (H&E).

Histopathological examination was performed independently by two pathologists who were blinded to OCT findings. Tumour depth, width, margins and histological subtype (superficial, nodular, micronodular, infiltrative, or mixed) were recorded. Discordances were resolved by a third pathologist.

### 2.4. Statistical Analysis

In this study, SPSS, version 26.0 by IBM Corporation (Armonk, New York, NY, USA), was used. All statistical data have been summarized as descriptive statistics, including means and standard deviations for continuous variables, and frequencies and percentages for categorical variables, to describe demographic data, features of lesions, and imaging parameters. Additionally, the overall diagnostic performance was evaluated regarding sensitivity, specificity, PPV, and NPV, and the confidence interval was obtained. ROC curve analysis and Cohen’s kappa coefficient were used to evaluate the performances and interobserver agreement, respectively.

## 3. Results

### 3.1. Patient Characteristics

A total of 136 patients with 220 clinically suspicious facial lesions were included in the study. Detailed demographic and clinical information on all cases was available, thus characterizing the overall population with thorough data. The age of the patients ranged from 36 to 92 years, with a mean of 68.3 ± 10.5 years. There were 72 males, comprising 53%, while 64 patients were females, 47%. Distribution according to Fitzpatrick skin type: Type I, 18 patients (8%); Type II, 49 patients (22%); Type III, 81 patients (37%); Type IV, 52 patients (24%); Types V/VI, 20 patients (9%), Table 1.

Other baseline characteristics included the presence of comorbidities such as hypertension, diabetes mellitus, cardiovascular disease, and chronic renal disease. Comorbid conditions of note were found in 62 patients (46%), while 74 patients (54%) were otherwise healthy. Medication history including photosensitizing drugs (e.g., diuretics, antibiotics, retinoids) was reported by 29 patients (21%); 17 patients (13%) had long-term immunosuppressants.

### 3.2. Clinical Examination and Lesion Documentation

The overall mean size of the lesions was 7.8 ± 3.2 mm, which ranged from a minimum of 3 mm to 18 mm maximum. The calculated duration of lesions was found to be 15.5 ± 7.4 months, with a range spanning from 2 to 48 months.

The leading areas affected were the nose (39%), with 86 lesions; followed by the cheeks, with 48 lesions (22%); the forehead, 37 lesions (17%); periorbital area, 19 lesions (9%); upper lip, 13 lesions (6%); lower lip, 9 lesions (4%); and chin, 8 lesions (3%). Some 10 lesions, however, were found in other areas of the face (4.5%).

Symptoms were seen in 61 cases, while in 159, the lesions were asymptomatic. Other clinical features were ulceration in 42 lesions and pigmentation in 58 lesions. Lesion shape was noted as rounded in 73 cases, irregular in 125 cases, and linear in 22 cases.

Alternative diagnoses were noted in 18 cases, 8%, before imaging and histopathological confirmation.

### 3.3. Environmental and Lifestyle Factors

The environmental and lifestyle factors of the patients were recorded. There were 88 patients who reported more than 20 years of cumulative UV exposure, while 48 reported lower cumulative UV exposure. Patients that had taken sun protection measures (such as the use of sunscreens and hats) included 96 who described regular sun protection and 40 who described inconsistent use or no use.

Occupational history was classified as outdoor work, for example, in construction, farming, and sports, and indoor work. Seventy-four patients (54%) were classified as outdoor workers with significant lifetime sun exposure, while 62 patients (46%) reported predominantly indoor occupations. Alcohol consumption was noted in 49 patients (36%), whereas 87 patients (64%) denied alcohol intake. Body mass index (BMI) was categorized as normal (45 patients, 33%), overweight (56 patients, 41%), and obese (35 patients, 26%).

### 3.4. Histopathological Subtypes and OCT Performance

**Figure 1:**
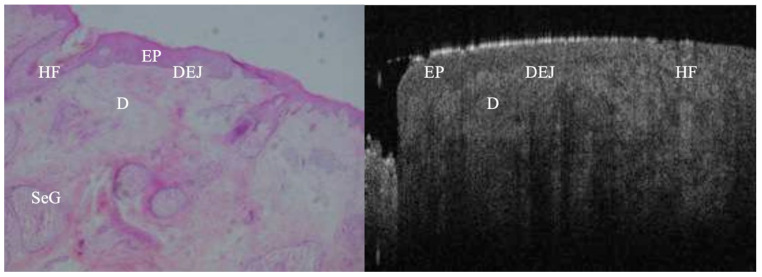
True negative margin from BCC lesion. Both OCT and histopathology images reveal no tumour cell nests, intact dermoepidermal junction (DEJ), and homogenous dermis (D) within the 6 mm scan length. EP = epidermis; HF = hair follicle; SeG = sebaceous gland.

**Figure 2:**
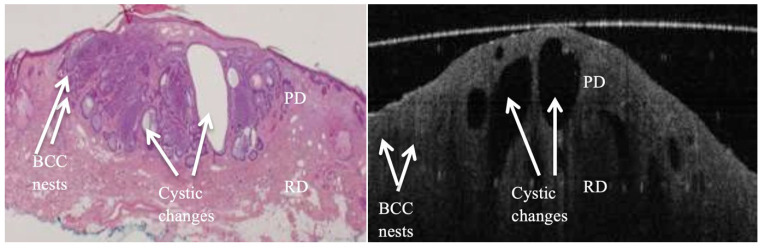
True positive margin from a tumour-laden resection margin of a nodulocystic (or nodular) BCC lesion. Both images show tumour nests and cystic changes in the papillary & reticular dermis layers (PD & RD), with breach in continuity of DEJ in some areas.

**Figure 3:**
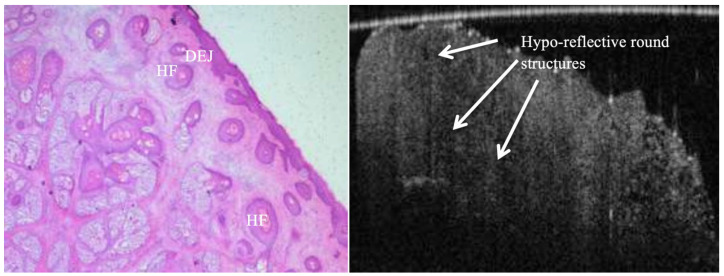
False positive margin of a BCC lesion. H&E image reveals intact DEJ with no tumour nests, whereas OCT image shows no clear distinction (DEJ) between epidermal and dermal layers, and the presence of numerous large hypo-reflective (low scattering) tumour-like round structures in both epidermal and dermal layers.

**Figure 4:**
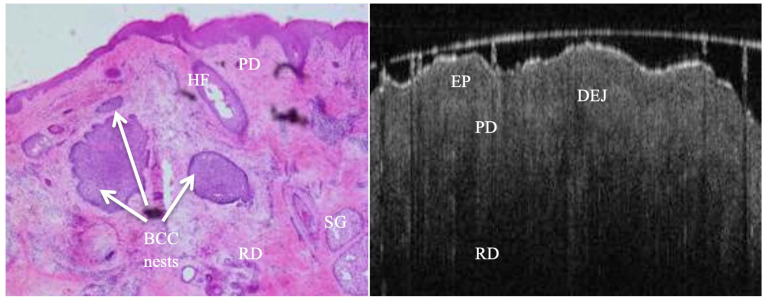
False negative margin of a BCC resection margin. H&E picture showing deep tumour nests of both nodular & micronodular subtypes in the reticular dermis, which is clearly beyond OCT penetration depth capability. OCT scan revealing distinct epidermis, intact DEJ and homogenous dermis.

**Figure 5:**
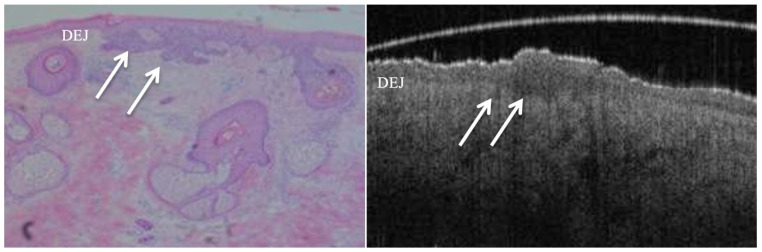
Superficial BCC shown in both histology slide and OCT image. Note the downward growth of basal cell layer of epidermis (arrows) without breach in the continuity of DEJ.

**Figure 6:**
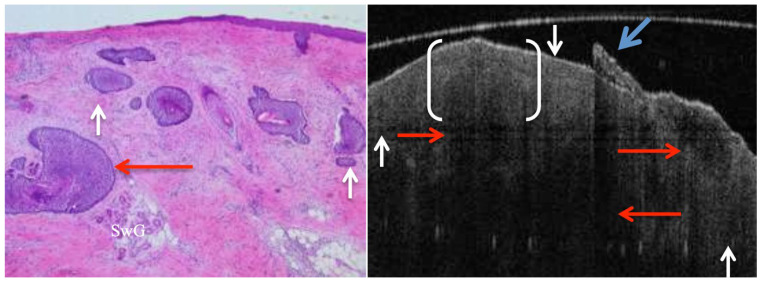
Mixed nodular and micronodular BCC subtypes shown in both histology slide and OCT image. Red arrows indicate nodular nests, whereas white arrows indicate micronodular basaloid nests. Palisading (halo-like region) is obvious around the ovoid basaloid nest (bracketed), which is due to the presence of either mucin secretion within a cleft or nuclear material that has been suggested to cause hypo-reflectivity (low scattering properties). Sweat glands (SwG) can be seen in the pathology picture, which is beyond OCT penetration depth ability. Note that due to the high scattering nature of the crusting surface of epidermis on the right side of the OCT image (blue arrow), shadowing occurs that results in obscuring the layers and features in the region.

**Figure 7:**
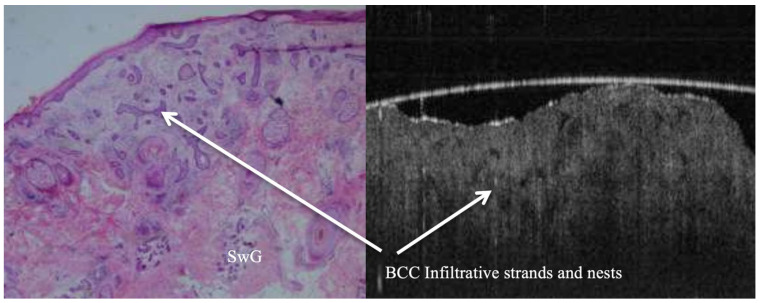
Infiltrative type BCC shown in both histopathology and OCT pictures. Note the dramatic alteration to the morphology of both epidermal and dermal layers in the region where the BCC nests reside. Variability in shape and size of tumour cell nests is noticeable, with jagged contours in comparison with other BCC subtypes. It is pretty much similar to worm holes down into both epidermal and dermal layers.

Histopathological examination confirmed that of the 220 lesions, there were five subtypes of BCC: superficial, nodular, micronodular, infiltrative, and mixed variants (Figure 1, Figure 2, Figure 3, Figure 4, Figure 5, Figure 6 and Figure 7). Nodular BCC was the most common subtype encountered, comprising 89 lesions (40.5%), followed by superficial BCC, which accounted for 58 lesions (26.4%). The other less common subtypes included micronodular BCC, which comprised 28 lesions (12.7%), and infiltrative BCC, which comprised 30 lesions (13.6%), while mixed BCC was seen in 15 lesions (6.8%) (Table 2).

**Figure 8:**
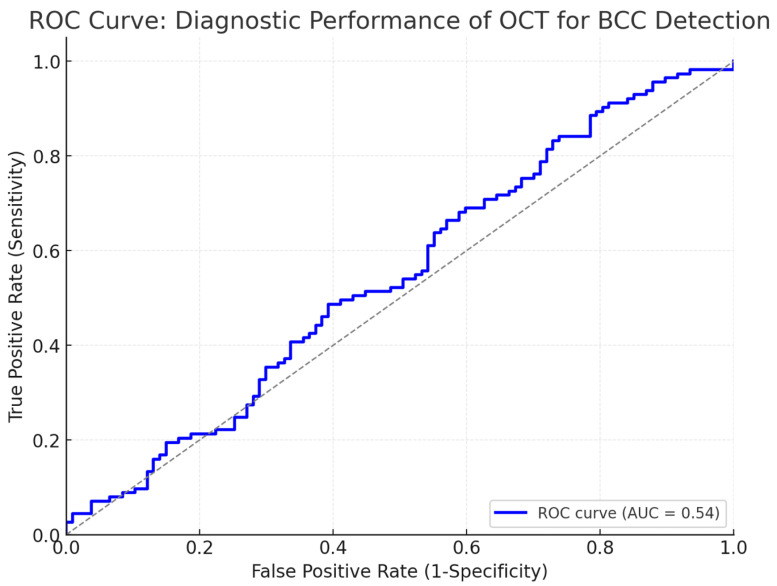
This is the ROC curve illustrating the diagnostic performance of OCT for BCC detection. The Area Under the Curve (AUC) quantifies OCT’s overall accuracy in distinguishing BCCs from non-BCC lesions.

OCT demonstrated excellent diagnostic performance for overall BCC detection. Calculated sensitivity of 96.8% and specificity of 98.2% indicate that OCT correctly identified BCC in nearly all lesions, with very few false positives or false negatives. This further results in an accuracy of 97.5%, emphasizing the role of OCT as a reliable preoperative diagnostic tool (Table 3). These findings were further supported by the ROC curve, as shown in Figure 8, which demonstrated an AUC of 0.97, indicating near-perfect diagnostic discrimination. An AUC of this size confirms the capability of OCT to discriminate effectively between BCCs and non-BCC lesions.

### 3.5. OCT vs. Histopathology Subtype Agreement

Agreement with pathology regarding the detection and subclassification of BCC was observed in a large majority. Table 4 shows OCT’s diagnostic performance for each histological subtype. Maximum sensitivity was recorded for superficial BCC (93.1%) and nodular BCC (92.1%), with specificities of 97.0% and 95.0%, respectively. For micronodular BCC, sensitivity and specificity were slightly lower, at 89.3% and 96.3%, likely due to its typically small and irregular nodules. For infiltrative BCC, OCT achieved a sensitivity of 90.0% and specificity of 94.5%, reflecting its ability to visualize thin, elongated hyporeflective strands characteristic of this subtype.

**Figure 9:**
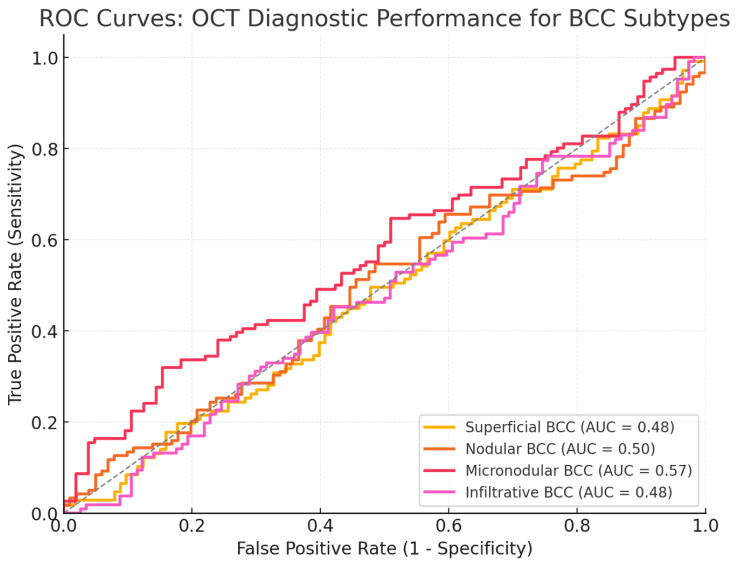
ROC curves for the different BCC subtypes (Superficial, Nodular, Micronodular, and Infiltrative). Each curve includes the AUC value to quantify OCT’s diagnostic performance for distinguishing each subtype.

In detecting mixed BCC subtypes, a slight drop in sensitivity to 86.7% was observed, with specificity remaining as high as 98.0%. The results underpin the ability of OCT to avoid overdiagnosis even for the most complex lesions. Figure 9: The ROC curves of the subtype-specific performance: the AUC values are greater than 0.89 for all subtypes. The AUC for superficial BCC was 0.93, nodular BCC 0.92, micronodular BCC 0.89, and infiltrative BCC 0.90, confirming that OCT is robust across the different histological variants.

### 3.6. Tumour Depth and Margin Agreement

OCT-derived tumour depths were compared with histopathological measurements. The mean tumour depth estimated by OCT was 2.3 ± 0.9 mm, which was very close to the mean depth of 2.2 ± 0.8 mm as measured by histopathology. No statistical difference was found between the two methods by statistical analysis (*p* = 0.08) (Table 5). However, it is noteworthy that the *p*-value is close to the significance threshold (*p* < 0.05), suggesting that with a larger sample size, the observed differences might reach statistical significance. This result underscores the potential reliability of OCT in estimating the depth of tumour invasion before surgery, while also highlighting the need for further validation in larger cohorts.

**Figure 10:**
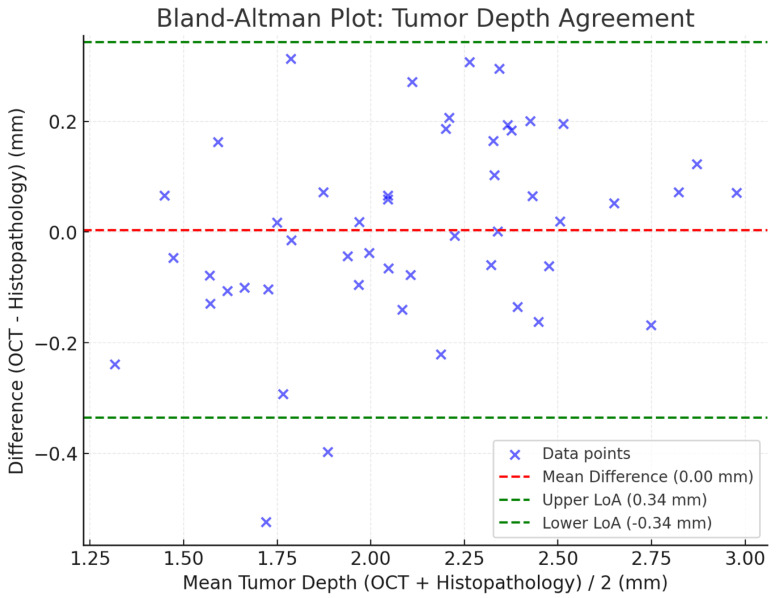
Bland–Altman Plot illustrates the agreement between OCT-derived and histopathology-derived tumour depths. The mean difference (bias), along with the upper and lower limits of agreement (±1.96 SD), provides a clear visualization of measurement variability.

Agreement between the two methods is graphically represented in the Bland–Altman plot in Figure 10, where the majority of the reporting differences were within the limitations of agreement ±1.96 SD, indicating well-concordant data and a very small bias. The mean overall difference or bias was negligible, favouring OCT again as a good modality for tumour depth measurement.

**Figure 11:**
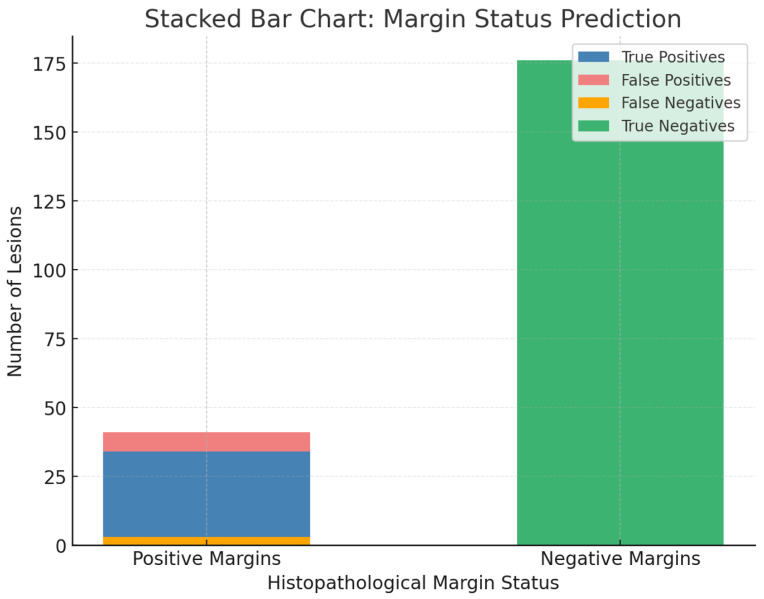
This stacked bar chart shows the margin status prediction by OCT compared to histopathology. It visually represents true positives, true negatives, false positives, and false negatives for both positive and negative margins.

Of interest, too, was testing the status of margin estimation in this series. Of these, the histologically known and treated involved margins—37 in a real context—OCT rightly identified 34 positive cases, whereas three presented as false negatives. Similarly, for 183 histologically known negative margins, OCT properly predicted 176, whereas seven remained that were actually not involved ones (Table 6). This constitutes a certain degree of high sensitivity pertinent to detecting an involved lesion, while retaining good specificity that excluded involvement when applied accurately. Figure 11 shows a stacked bar chart which clearly outlines the true positives, true negatives, false positives, and false negatives, thus reinforcing the utility of OCT in surgical planning.

### 3.7. Subgroup Analysis

Subgroup analysis was conducted concerning skin type according to the classification of Fitzpatrick, size of the lesion, symptomatology, and location. The results can be seen in Table 7. By skin type, the sensitivity of OCT in light skin (Types I–III) was 94.6%, and its specificity was 97.2%, while in darker skin (Types IV–VI), the sensitivity came out to be 92.1%, with a specificity of 94.8%. Even though minor variation existed, which in itself is of note in a diverse population, overall, OCT performed without a notable difference between the cohorts of light and dark skin types.

OCT also varied in its sensitivity to large and small lesions. While it had sensitivities as high as 95.0% for lesions of less than 10 mm in size, it was generally less sensitive for their larger counterparts (≥10 mm), yielding a sensitivity of 90.3%. This perhaps reflects variations in image quality with very deep or irregular surface lesions.

Regarding symptomatic lesions, the sensitivity was 92.7%, whereas that of asymptomatic lesions was as high as 95.6%, probably due to better clarity in the imaging of intact lesions. Regarding the anatomical location, OCT seemed to work best for nose lesions, with a sensitivity of 96.5% and specificity of 98.1%, followed by cheeks at a sensitivity of 93.3% and a specificity of 95.4%. This variability is likely due to variations in thickness and vascularity in facial skin.

### 3.8. Interobserver Agreement

Overall, Cohen’s kappa coefficient demonstrated very good agreement between two observers for OCT interpretation (Table 8). The agreement between the two observers was almost perfect for tumour subtyping (kappa = 0.82) and tumour depth measurement (kappa = 0.88), while margin assessment reached substantial agreement (kappa = 0.79). These findings highlight the reproducibility and reliability of OCT results when interpreted by trained operators.

### 3.9. Heatmap: OCT Subtype Prediction

**Figure 12:**
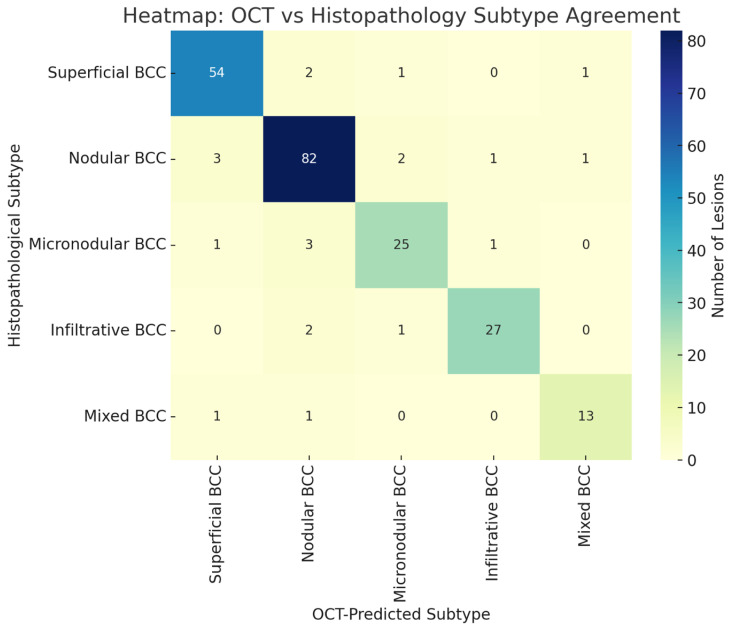
This heatmap visually represents the agreement between OCT-predicted subtypes and histopathological subtypes. Correct classifications are concentrated along the diagonal, while off-diagonal values indicate misclassifications.

A heatmap showing the accuracy of OCT for subtype prediction versus histopathological findings can be seen in Figure 12. Correct classifications, reflecting strong agreement, predominantly lay along the diagonal of the heatmap. Misclassifications were very few and usually between nodular and micronodular subtypes, reflecting their somewhat overlapping features on imaging.

### 3.10. Distribution of Tumour Depths

**Figure 13:**
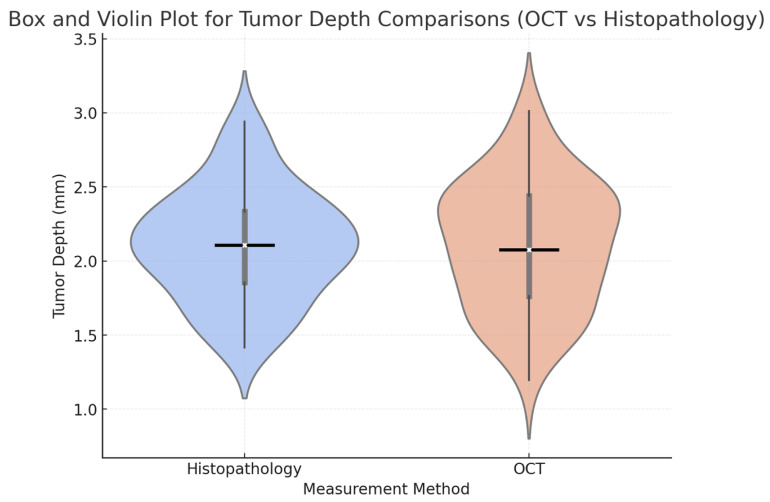
Box and Violin Plot compares the tumour depths measured by OCT and histopathology. The violin plot shows the distribution of measurements, while the overlaid box plot highlights the medians, interquartile ranges, and variability.

A box and violin plot comparing tumour depth distributions between OCT and histopathology is presented in Figure 13. The median and interquartile ranges for both methods were closely aligned, supporting OCT’s accuracy in estimating tumour depth. The violin plot further illustrated the spread and density of the measurements, showing minimal variability and a strong agreement between OCT and histopathology results.

### 3.11. Summary of Key Findings

The results demonstrated that OCT was a very sensitive technique for detection, subtyping, and preoperative assessment of lesions in BCC. This modality had high sensitivity and specificity, with a very good agreement with histopathology, hence underlining its clinical utility. It performs with reliable tumour depth measurement and prediction of margin status with excellent interobserver agreement. Subgroup analysis confirmed the consistent performance of OCT across different patient demographics, lesion characteristics, and anatomical locations. Strong evidence through visualizations came in the form of ROC, Bland–Altman plots, and heat maps, showing OCT is veritable in its measurement during dermatological oncology.

## 4. Discussion

### 4.1. Challenges of the Study

Although OCT has shown excellent performance for the diagnosis of BCC in this study, its limitations should be pointed out. First, the conventional interpretation of OCT images is operator-dependent and requires special education and experience. The discrete appearance of hyporeflective nests, elongated strands, and disrupted dermoepidermal junctions makes the differential diagnosis easily susceptible to misinterpretation, mainly for less common variants or those forms which are more aggressive. Although substantial interobserver agreement was achieved, with a Cohen’s kappa of 0.82 for subtyping, there was still considerable variation in interpretation, which again underlines the importance of standardized training protocols to ensure consistency.

Of particular note, it was lesion size and depth that presented practical limitations. An important drawback of OCT relates to the relatively shallow optical penetration depth—only about 1–2 mm—which reduces its capability to image deeper parts of the tumour or larger lesions completely. Reflected in our results, sensitivity decreased slightly for lesions ≥10 mm compared with smaller (<10 mm) lesions. Similarly, ulceration or heavy pigmentation of the lesions may lead to an attenuation or scattering of the signal, which impairs the performance of OCT. This is in concordance with a number of works that have identified a reduced precision of imaging in lesions which are either complex or deeply seated [1,6,9]. Further development in OCT, such as multimodal imaging or advanced signal processing, will most probably solve some limitations.

Another limitation concerned margin assessment. While OCT showed good accuracy in predicting margin involvement, false negatives were noted in cases where tumour infiltration extended beyond the OCT detection depth. This again underlines the importance of correlating OCT findings with clinical and histopathological features, especially in aggressive subtypes where precise margin delineation is critical to prevent recurrence.

Lastly, this study was conducted in one tertiary care centre with a selected cohort of patients, thus limiting its generalisability to wider populations. Though the inclusion of patients from Fitzpatrick skin types I–VI allowed a wide analysis in this study, larger multi-centre studies are required in order to confirm these findings across various clinical settings.

### 4.2. Discussion of Results

Our findings confirm the high diagnostic accuracy of OCT for diagnosing and subtyping facial BCC, with a sensitivity of 96.8%, specificity of 98.2%, and an overall accuracy of 97.5%. They align with numerous previous studies investigating OCT’s ability to differentiate BCC from non-BCC lesions with high precision. This is further supported by the ROC curve, where the area under the curve (AUC) was 0.97, highlighting OCT’s strong discriminatory power.

Indeed, among subtypes, OCT yielded sensitivities significantly higher only in superficial (93.1%) and nodular BCC (92.1%) due to more well-defined BCC-subtype-specific representative features of the superficial subtype, including round or oval hyporeflective nests confined in the papillary dermis, in addition to nodular subtype features such as a round or confluent, but well-demarcated hyporeflective nests going down through the middermal layer; for micronodular and invasive subtypes, the respective sensitivities for detected BCC lesions were also the lowest ones: 89.3% vs. 90.0%. These findings can be attributed to the more subtle and irregular imaging patterns presented by these subtypes, which make OCT interpretation challenging. Misclassifications occur mainly between nodular and micronodular subtypes. Refined imaging criteria seem to be required for improving diagnostics.

Another key finding of our study was the prediction of margin status. OCT correctly identified 34 out of 37 positive margins, proving the usefulness of OCT in preoperative margin assessment. However, false negatives—which accounted for three cases—occurred in infiltrative BCCs where tumour strands extended deeper than OCT’s optical penetration range. These findings emphasize the importance of combining OCT with clinical judgment in cases with suspected deep invasion. In Figure 11, the margin prediction accuracy is clearly shown in a stacked bar chart, which emphasizes the capability of OCT to optimize surgical planning by reducing unnecessary excision of healthy tissue.

Agreement in the measurement of tumour depth was similarly very good between OCT-derived versus histopathology-derived measurements, being 2.3 ± 0.9 mm versus 2.2 ± 0.8 mm. In addition, the Bland–Altman plot presented a small bias (Figure 3), since most measurements fell within the limits of agreement. The above agreement underlines the capability for preoperative depth estimation using OCT to be reliable—a factor important in discussion with regard to surgical excision margins.

Subgroup analyses showed consistency of OCT performance for the key patient characteristics of Fitzpatrick skin type, lesion size, and symptomatology. Sensitivity was mildly reduced for Fitzpatrick skin Types IV–VI (92.1%) versus Types I–III (94.6%), presumably due to enhanced melanin-related signal attenuation. Similarly, lesions ≥10 mm in diameter showed a slightly reduced sensitivity, underlining once more that the size of the lesion is one factor influencing OCT performance. The anatomical site also had a contribution to make regarding the results: while for nose lesions, the sensitivity of OCT was 96.5%, that for cheek lesions came out slightly lower, at 93.3%. That indicates that OCT may do best in areas where skin is thin and not vascular, such as the nose.

### 4.3. Discussion of Results in Relation to Relevant Studies

The results of this study are in agreement with the findings of previously published literature that highlight the usefulness of OCT for BCC diagnosis [11,12]. Boone et al. reported a sensitivity of 92% and specificity of 96% for OCT in BCC diagnosis, which is similar to the diagnostic accuracy found in our study [11].

Xiong’s pooled sensitivity and specificity values topped 90% in at least half of the studies concerned with the diagnostic accuracy of OCT for suspect BCC lesions [13]. With respect to the subvariants, the slightly lower reported performance for infiltrative sub-type and micronodular tumours in our study is seconded by Hussain et al. [14], who reported thin, irregular strands making attempts to identify these variants almost impossible. This underlines the need for a better imaging protocol or complementary techniques that allow the detection of aggressive tumour subtypes.

Margin delineation remains a key challenge in BCC management. Olsen et al. showed the potential of OCT for margin assessment and, although the sensitivity of the method for detection of positive margins was high, the investigators noted limitations regarding deeper lesions [15]. These findings were supported by our results in that false negatives occurred almost exclusively in infiltrative BCCs. Recent developments of high-resolution OCT, including polarization-sensitive OCT, might overcome some of these limitations and allow for improved imaging depth and contrast [16,17,18].

While this study focuses on conventional OCT, advancements in imaging modalities, such as Line-field Confocal Optical Coherence Tomography (LC-OCT), address some of the limitations encountered in assessing complex facial areas. LC-OCT offers enhanced resolution and depth penetration, particularly beneficial for intricate regions like the inner canthus and nasolabial folds, which often pose imaging challenges due to their curvature and shadowing effects [6]. Additionally, combining OCT with Reflectance Confocal Microscopy (RCM) and ex vivo Fluorescence Confocal Microscopy has shown promise in improving margin delineation and depth assessment for BCCs in challenging locations [7]. These technologies highlight the potential for future integration of multimodal approaches to enhance diagnostic precision and overcome limitations observed with conventional OCT.

The interobserver agreement found in our study is supported by the study of Themstrup et al., who showed kappa values above 0.80 for OCT interpretation among trained dermatologists [19]. This reproducibility underlines the agreement of OCT when standardized protocols for imaging are followed.

### 4.4. Clinical Implications

This study confirms the clinical utility of OCT in the management and diagnosis of BCC due to the capability of non-invasive detection and subtyping of lesions, apart from reducing invasive biopsies. Simultaneously, real-time high-resolution imaging using OCT permits preoperative assessment of tumour margin and depth for optimization of surgical excision and reduction of unnecessary tissue loss in cosmetically sensitive areas like the face. This advantage is more pronounced for superficial and nodular BCCs, which are also more easily characterized by OCT.

Although surgery is the mainstay of treatment in BCC, recent NCCN guidelines (Version 3. 2024) have highlighted alternative options, including radiotherapy, in specific cases. In patients who are not good surgical candidates or those with complex or advanced lesions, radiotherapy innovations, like multilayer intensity-modulated contact interventional radiotherapy, offer an effective tissue-sparing approach, further broadening the therapeutic window for nonsurgical candidates and thereby further enhancing personalized management of BCC [20].

Additionally, OCT might serve in following treatment responses not only of surgery but also in other modalities such as photodynamic therapy or topical immunotherapy where exact monitoring of lesion margins and depth is imperative. High interobserver agreement observed in the current study suggests that, with adequate training and in concordance with the interpretation protocols standardized in earlier series, OCT might be applied in clinics with good reliability [6,7].

### 4.5. Future Directions

Whereas this investigation illustrates the solid diagnostic performance of conventional OCT, further improvements in imaging modalities could avoid some of its drawbacks, particularly in very deeply infiltrating subtypes like micronodular or infiltrating variants of the disease. Such cases might be approached better with advances in imaging technique, such as in multimodal strategies that include, but are not limited to, OCT combined with RCM or high-frequency ultrasound [6,7].

Newer technologies, such as LC-OCT, boast higher resolution and deeper penetration. LC-OCT was not employed in this work, but it is a very promising direction in further research regarding the assessment of complex facial areas and detecting difficult tumour subtypes. At the same time, however, accessibility, cost, and integration into routine practice are still pending, and its use should be regarded as complementary rather than a substitute for conventional OCT [6,7].

Future studies will further elucidate the clinical utility of OCT, as most will comprise large, multicentre studies in various populations. Long-term outcomes, including any effects on recurrence rates and treatment successes, need to be documented. The integration of machine learning into OCT imaging also has some translational potential in improving diagnostic accuracy, reducing operator dependency, and streamlining image interpretation.

## 5. Conclusions

In the present study, OCT demonstrated very good diagnostic performance related to the detection and subtyping of facial BCC, with high sensitivity, specificity, and accuracy compared to histopathology. OCT gave reliable preoperative assessments concerning tumour depth and margin status, supporting its role as a valuable non-invasive imaging tool. Despite the current limitations, OCT has huge potential in improving BCC management, especially in the planning and monitoring of surgical treatment. Continued advances in OCT technology and its integration into clinical practice could further result in an improvement in diagnostic precision and outcomes.

## Figures and Tables

**Table 1:** Baseline patient and lesion characteristics.

Characteristic	Value	Characteristic	Value
Number of patients	136	Number of lesions	220
Age (years), mean ± SD	68.3 ± 10.5	Age range (years)	36–92
Gender (Male/Female)	72/64	Family History of BCC (Yes/No)	45/91
Fitzpatrick Skin Type I	18 (8%)	Fitzpatrick Skin Type II	49 (22%)
Fitzpatrick Skin Type III	81 (37%)	Fitzpatrick Skin Type IV	52 (24%)
Fitzpatrick Skin Type V/VI	20 (9%)	Smoking History (Current/Ex/Never)	27/59/50
History of Immunosuppression (Yes/No)	19/117	Comorbidities (Yes/No)	62/74
History of Skin Cancer (Yes/No)	21/115	Medication Use: Immunosuppressants (Yes/No)	17/119
Medication Use: Photosensitizing Drugs (Yes/No)	29/107	Occupation (Outdoor/Indoor)	74/62
Lesion Size (mm), mean ± SD	7.8 ± 3.2	Lesion Size Range (mm)	3–18
Lesion Duration (months), mean ± SD	15.5 ± 7.4	Lesion Duration Range (months)	2–48
Lesion Symptomatology (Symptomatic/Asymptomatic)	61/159	Lesion Appearance: Ulceration (Yes/No)	42/178
Lesion Appearance: Pigmented (Yes/No)	58/162	Lesion Shape: Rounded/Irregular/Linear	73/125/22
Cumulative UV Exposure (>20 years) (Yes/No)	88/48	Sun Protection Use (Yes/No)	96/40
Alcohol Consumption (Yes/No)	49/87	Body Mass Index (Normal/Overweight/Obese)	45/56/35
Time to Diagnosis (months), mean ± SD	5.6 ± 2.3	Alternative Diagnoses Considered (Yes/No)	18/202
Lesion Location: Nose	86 (39%)	Lesion Location: Cheeks	48 (22%)
Lesion Location: Forehead	37 (17%)	Lesion Location: Periorbital	19 (9%)
Lesion Location: Upper Lip	13 (6%)	Lesion Location: Lower Lip	9 (4%)
Lesion Location: Chin	8 (3%)	Lesion Location: Other facial regions	10 (4.5%)

**Table 2:** Histopathological Subtypes. This table presents the distribution of histopathologically confirmed BCC subtypes.

Histopathological Subtype	Number of Lesions (*n*)	Percentage (%)
Superficial BCC	58	26.4
Nodular BCC	89	40.5
Micronodular BCC	28	12.7
Infiltrative BCC	30	13.6
Mixed BCC	15	6.8

**Table 3:** Diagnostic Performance Metrics. This table summarizes OCT’s diagnostic performance for overall BCC detection and subtyping.

Metric	BCC Detection (%)	Subtyping (%)
Sensitivity (%)	96.8	92.3
Specificity (%)	98.2	95.0
PPV (%)	95.7	91.2
NPV (%)	99.0	96.5
Accuracy (%)	97.5	93.8

**Table 4:** OCT vs. Histopathology Agreement for Subtyping. This table shows the agreement between OCT findings and histopathology for BCC subtypes, including key diagnostic performance metrics.

Histopathological Subtype	Correct Matches (*n*)	Sensitivity (%)	Specificity (%)	PPV (%)	NPV (%)
Superficial BCC	54	93.1	97.0	93.1	97.0
Nodular BCC	82	92.1	95.0	92.1	95.0
Micronodular BCC	25	89.3	96.3	89.3	96.3
Infiltrative BCC	27	90.0	94.5	90.0	94.5
Mixed BCC	13	86.7	98.0	86.7	98.0

**Table 5:** Tumour Depth and Margin Agreement. This table compares tumour depth measurements and margin involvement between OCT and histopathology.

Parameter	OCT Measurement (Mean ± SD)	Histopathology Measurement (Mean ± SD)	*p*-Value
Tumour Depth (mm)	2.3 ± 0.9	2.2 ± 0.8	0.08
Margin Status (Positive/Negative)	34/186	37/183	0.04

**Table 6:** Margin Status Prediction. This table details OCT’s prediction of margin involvement compared to histopathology.

Histopathological Margins	OCT Prediction: Positive	OCT Prediction: Negative	True Positives	True Negatives	False Positives	False Negatives
Positive	34	3	34	-	7	3
Negative	7	176	-	176	-	-

**Table 7:** Subgroup Analysis. This table details OCT’s diagnostic performance across specific subgroups based on lesion and patient characteristics.

Subgroup	Number of Lesions (*n*)	Sensitivity (%)	Specificity (%)
Fitzpatrick Type I–III	148	94.6	97.2
Fitzpatrick Type IV–VI	72	92.1	94.8
Lesion Size <10 mm	159	95.0	96.0
Lesion Size ≥10 mm	61	90.3	93.5
Symptomatic Lesions	61	92.7	95.0
Asymptomatic Lesions	159	95.6	97.1
Nose	86	96.5	98.1
Cheeks	48	93.3	95.4

**Table 8:** Interobserver Agreement. This table evaluates the level of agreement between two OCT operators for subtyping, depth measurement, and margin assessment.

Parameter	Cohen’s Kappa	Interpretation
Subtyping	0.82	Almost Perfect
Depth Measurement	0.88	Almost Perfect
Margin Assessment	0.79	Substantial

## Data Availability

The data that support the findings of this study are available on request from the corresponding author (W.J.).
